# Elucidating the isorhamnetin-3-O-glucoside-iNOS interaction via molecular dynamics and Hirshfeld surface analyses

**DOI:** 10.1371/journal.pone.0339357

**Published:** 2025-12-19

**Authors:** Oussama Khibech, Haytham Bouammali, Yousra Hammouti, Mohamed bouhrim, Mohammed Merzouki, Said Abadi, Mohammed Al-zharani, Fahd A. Nasr, Ashraf Ahmed Qurtam, Boufelja Bouammalli, Allal Challioui

**Affiliations:** 1 Laboratory of Applied Chemistry and Environment (LCAE), University Mohammed Premier, Faculty of Science, Oujda, Morocco; 2 Laboratoire d’Amélioration des Productions Agricoles, Biotechnologie et Environnement (LAPABE), Faculté des Sciences, Université Mohammed Premier, Oujda, Morocco; 3 Laboratoires TBC, Laboratory of Pharmacology, Pharmacokinetics, and Clinical Pharmacy, Faculty of Pharmaceutical and Biological Sciences, Lille, France; 4 Biology Department, College of Science, Imam Mohammad Ibn Saud Islamic University (IMSIU), Riyadh, Saudi Arabia; Universidade Federal do Para, BRAZIL

## Abstract

Inducible nitric oxide synthase (iNOS) remains a demanding metallo-enzyme target because the catalytic heme shapes both geometry and electrostatics at the binding site. We evaluated the dietary flavonol glycoside isorhamnetin-3-O-glucoside (I3OG) against mouse (3E6T) and human (3E7G) iNOS oxygenase domains using a heme-aware, auditably validated docking workflow. we centered the docking grids at the crystallographic Fe position and validated the protocol by re-docking the native co-crystallized inhibitors (3E6T: AR-C118901/1A2; 3E7G: AR-C95791/AT2), reproducing the crystal poses with heavy-atom RMSD = 1.093 Å and 0.327 Å, respectively (≤ 2.0 Å criterion). Explicit-solvent 100-ns MD confirmed stable complexes for both systems; 3E6T showed tighter ligand RMSD, lower pocket Cα-RMSF, and a more persistent H-bond network. MM/GBSA over equilibrated frames (60−100 ns) yielded ΔG_bind ≈ −44.9 ± 3.9 kcal·mol^−1^ (3E6T) vs −36.1 ± 3.7 kcal·mol^−1^ (3E7G), with per-residue hot spots matching docking contacts. Principal-component free-energy maps indicated more focused minima for 3E6T and a broader low-energy valley for 3E7G, consistent with the MD metrics. we performed an apo-form heme-cavity test (heme removed, grid kept at Fe; proximal Cys re-protonated) to probe pocket occupancy/flexibility without claiming a catalytic model. Collectively, the heme-centred, co-crystal-validated protocol plus the apo-cavity readout support I3OG as a plausible scaffold for iNOS engagement and provide a transparent template for future metallo-enzyme docking studies.

## 1. Introduction

Inducible nitric oxide synthase (iNOS) is a central enzymatic source of high-output nitric oxide (NO) in innate and adaptive immunity, where dysregulated NO contributes to chronic inflammation, metabolic and cardiovascular disorders, and cancer [1–4]. Therapeutic modulation of iNOS remains attractive yet challenging because selectivity and on-target efficacy must be reconciled with complex redox biochemistry and multi-domain conformational control. Structural and biochemical studies have mapped the oxygenase domain, heme-pterin chemistry, and dimerization interfaces that govern activity, offering tractable footholds for ligand design and mechanism-guided inhibition [5].

Natural products especially dietary flavonoids have long been recognized for anti-inflammatory actions that include dampening NF-κB signaling and suppressing COX-2 and iNOS expression [6–8]. Within this class, isorhamnetin and its glycosides (notably isorhamnetin-3-O-glucoside, I3OG) show antioxidant and anti-inflammatory effects and reduce NO overproduction in cellular models, making them compelling chemical probes for iNOS modulation [9–11]. Recent surveys of isorhamnetin glycosides underscore their prevalence, pharmacology, and relevance to human health, while broader analyses emphasize the enduring role of natural products as leads for drug discovery [12].

Computational pipelines that combine carefully validated docking with explicit-solvent molecular dynamics (MD) and end-point binding free-energy calculations have become standard to interrogate protein-ligand recognition at atomistic resolution. Docking engines such as AutoDock Vina guide pose generation and enrichment [13,14], and learning-augmented rescoring further improves pose quality while retaining the classical RMSD ≤ 2.0 Å benchmark for pose fidelity [15,16]. Production MD implemented in modern GPU-accelerated codes captures conformational adaptation, ligand stability, and the time-dependent behavior of key observables (RMSD, RMSF, hydrogen bonds, and radius of gyration) [17–21]. Post-processing with MM/GBSA consolidates nonbonded interactions and solvation to estimate relative binding affinities and helps prioritize poses consistent with the dynamics [22,23].

Crucially, experimental crystal-packing analyses complement solution-phase simulations by mapping short-range contacts, π-stacking, and hydrogen-bonding motifs that underlie solid-state stability. Hirshfeld surface analysis and fingerprint plotting (as implemented in CrystalExplorer) provide a quantitative picture of intermolecular interactions that can be related to recognition patterns seen in complexes [24,25].

In this context, we investigate I3OG against iNOS oxygenase domains represented by the mouse (PDB 3E6T) and human (PDB 3E7G) structures [5,26]. We pair validated docking with long-timescale MD to monitor structural stability (RMSD), residue-level flexibility (RMSF), hydrogen-bond persistence, and global compaction (Rg), and we quantify binding via MM/GBSA using extensive trajectory snapshots to reduce statistical noise. Our structural choices leverage high-resolution templates and contemporary simulation protocols to minimize methodological bias and to ensure that free-energy trends are grounded in physically realistic dynamics.

iNOS is a validated yet challenging target in chronic inflammation and oncology, so robust, mechanism-aware leads are needed. We focus on I3OG because flavonol glycosides combine a favorable safety profile, tractability, and anti-inflammatory potential. Our objectives are to define stable binding modes of I3OG in murine and human iNOS oxygenase domains, quantify their relative binding free energies with MM/GBSA using dense sampling, and relate dynamic observables (RMSD, RMSF, hydrogen bonds, Rg) to energetic trends to strengthen biological plausibility. We also integrate crystal-packing insights (Hirshfeld) to cross-validate interaction motifs across solution and solid phases.

## 2. Materials and methods

### 2.1 Hirshfeld surface analysis with CrystalExplorer

In this study, we limited the Hirshfeld surface analysis to our crystallized compound only, Isorhamnetin-3-O-glucoside (I3OG). The crystallographic information file (CIF) was retrieved from the Crystallography Open Database (COD 1551703). CrystalExplorer 21.5 was applied to the compound’s CIF file to compute the descriptors d_norm_, d_i_, d_e_, Shape Index, and Curvedness, and to generate 2D fingerprint plots, allowing us to identify close contacts within the crystal packing and quantify the contributions of H···H, O···H/H···O, C···H/H···C interactions [24]. This crystallographic analysis complements our solution-phase results (MD/MM-GBSA), providing a coherent, multiscale picture of interaction patterns and system stability.

### 2.2 AutoDock Vina

**Fig 1:**
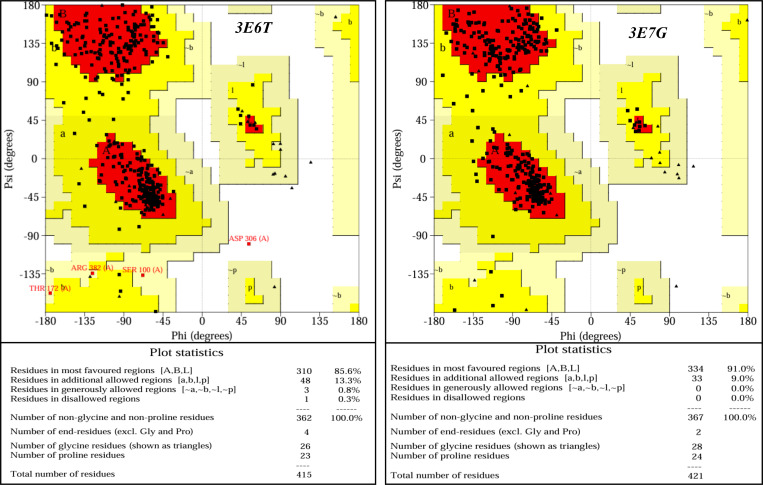
PROCHECK Ramachandran plots for iNOS oxygenase domains 3E6T and 3E7G (black squares = non-Gly/Pro; triangles = Gly). Regions: most-favoured (dark red), additionally allowed (orange), generously allowed (yellow), and disallowed (white).

Molecular docking was carried out with AutoDock Vina (via the ADT 1.5.7 interface) [27]. Protein structures (PDB IDs 3E6T and 3E7G) were preprocessed by removing crystallographic waters, adding polar hydrogens, and assigning charges following the AutoDock recommendations; Discovery Studio was used for inspection and minor corrections before and after docking. Before docking, the stereochemical quality of both receptors was verified with PROCHECK Ramachandran plots (Fig 1), confirming sound backbone geometry and allowing the native coordinates to be used without further remodeling. For 3E6T, 310 residues (85.6%) fell in the most favoured regions and 99.7% of non-glycine/non-proline residues were located in allowed regions, with only 1 residue (0.3%) in disallowed regions; for 3E7G, 334 residues (91.0%) were in the most favoured regions and 100% of residues were in allowed regions with no outliers.

**Fig 2:**
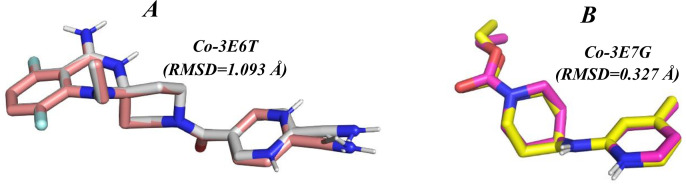
Redocking validation at the native heme site. Superposition of co-crystallized ligands and their re-docked poses for **(A)** 3E6T/1A2 (RMSD = 1.093 Å) and **(B)** 3E7G/AT2 (RMSD = 0.327 Å). Heavy-atom RMSD values were computed in PyMOL.

Ligands were sketched and geometry-optimized in ChemDraw, then converted to **PDBQT** with ADT to define torsions and charges. For the search space, the docking grid was centered at the crystallographic Fe of the heme in each structure. For 3E6T, the grid center was (123.93, 111.71, 32.17) Å with dimensions 17.30 × 13.89 × 21.57 Å; for 3E7G, the center was (57.69, 20.58, 84.96) Å with dimensions 15.44 × 16.81 × 16.93 Å. Docking used AutoDock Vina 1.1.2 with exhaustiveness = 8, num_modes_ = 8, and energy_range_ = 4; other settings were defaults. Poses were ranked by Vina score and inspected in ADT and Discovery Studio to assign hydrogen-bond and hydrophobic contacts. Protocol validation retained the heme as part of the receptor and re-docked the native co-crystallized inhibitors 3E6T: AR-C118901 (ligand code 1A2) and 3E7G: AR-C95791 (ligand code AT2) using the same grids. The best poses reproduced the crystal conformations with heavy-atom RMSD = 1.093 Å (3E6T/1A2) and 0.327 Å (3E7G/AT2) (computed in PyMOL), both well below the 2.0 Å criterion; Fig 2 shows the redocking overlays.

### 2.3 Implementation of molecular dynamics simulations using GROMACS

Molecular-dynamics simulations were performed with GROMACS 2021.3 [28]. Using gmx pdb2gmx with the AMBER99SB-ILDN force field, missing protein hydrogens were added and protonation states adjusted. The ligand was parameterised separately; the validated.itp and.gro files were then merged with the protein to build the full complex. This complex was centred in a cubic TIP3P water box, neutralised with counter-ions, and relaxed via steepest-descent energy minimisation. Sequential NVT and NPT equilibration phases stabilised temperature and pressure, respectively. Finally, a 100 ns production run was conducted, saving coordinates and velocities at regular intervals to characterise stability, conformational dynamics, and key intermolecular interactions under near-physiological conditions.

### 2.4 MM/GBSA calculation

Binding free energies were calculated with AmberTools23 (MMPBSA.py, parallel mode) using 100 snapshots sampled every 0.4 ns from the 60–100 ns window of each GROMACS trajectory. The HCT generalized-Born model (igb = 5) was applied with dielectric constants ε_in = 1.0 and ε_out = 80.0 and an ionic strength of 0.15 M. The nonpolar solvation term was estimated from the solvent-accessible surface area. Temporary files were not retained (keep_files = 0). Per-residue decomposition (idecomp = 1) was enabled to resolve van der Waals, electrostatic, polar, and nonpolar contributions for each residue.

### 2.5 DFT methods

All quantum-chemical calculations were carried out for the isolated ligands in the gas phase using ORCA 5.0.4, treating each molecule in its neutral singlet state (q = 0, multiplicity = 1). Ground-state geometries were optimized at the B3LYP-D3(BJ)/def2-SVP level, employing the RIJCOSX approximation with the def2/J auxiliary basis, TightSCF convergence criteria and an integration Grid5 [29]. Harmonic frequency analyses were performed to ensure that all optimized structures correspond to true minima (no imaginary frequencies). Frontier molecular orbital energies (E_HOMO_, E_LUMO_) were extracted from the converged SCF wavefunctions and used to derive conceptual-DFT descriptors according to: Egap = E_LUMO_ − E_HOMO_, η = Egap/2, S = 1/η, μ = (E_HOMO_ + E_LUMO_)/2, and ω = μ²/(2η).

HOMO/LUMO isosurfaces were generated from the optimized geometries and visualized in Avogadro 1.2.0 at a fixed isovalue (0.01 a.u.), using identical display settings for all ligands to enable direct cross-comparison.

## 3. Results and discussion

### 3.1 Hirshfeld-Surface

#### 3.1.1 Hirshfeld surface metrics and lattice-water-mediated crystal packing of I3OG.

Hirshfeld surface analysis provides a standardized way to connect the crystal structure to the nature of intermolecular contacts: it highlights short contacts on the d_norm_ map, describes surface topography via shape index and curvedness, and supplies concise morphological descriptors (surface volume, area, globularity, asphericity) that help rationalize crystal packing and interaction diversity. For isorhamnetin-3-O-glucoside, the calculations yielded a volume of 514.30 Å³ and a surface area of 440.60 Å², reflecting a relatively large molecular envelope for an aryl glycoside and indicating ample regions available for intermolecular contact [30]. A globularity of 0.705 (where 1 denotes a perfect sphere) points to a moderately globular shape neither rod-like nor flat so contacts are expected to be distributed over the surface rather than concentrated at one end. The asphericity of 0.114 (zero for a perfect sphere) is low, indicating only a modest departure from sphericity with slight elongation along the aromatic core and sugar [31]. Taken together, these descriptors suggest that the I3OG surface is well suited to form a mixed network of polar (H···O/O···H) and hydrophobic contacts, consistent with the picture to be refined by detailed Hirshfeld maps and the docking results.

**Fig 3:**
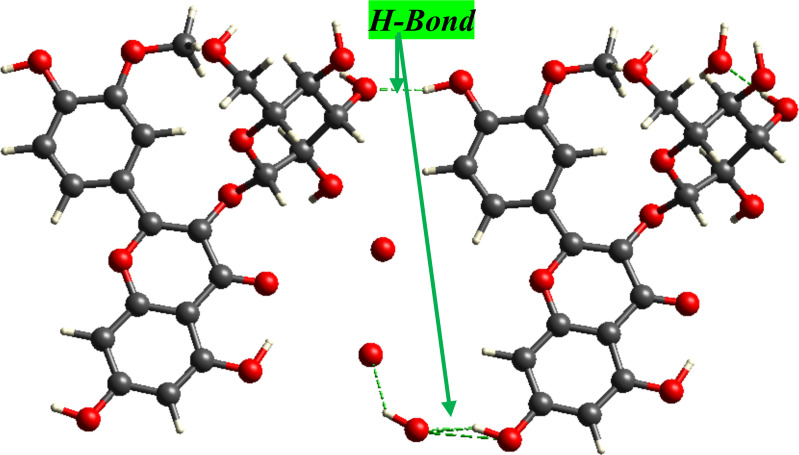
Lattice-water-mediated hydrogen bonding between neighboring isorhamnetin-3-O-glucoside molecules in the crystal.

The Fig 3 shows a hydrogen-bond bridge (green) linking neighboring isorhamnetin-3-O-glucoside molecules, mediated by lattice water. Dashed contacts indicate O-H···O interactions between ligand oxygen donors/acceptors that connect adjacent molecules. These intermolecular H-bonds help stabilize the crystal packing and anticipate strong O···H/H···O contributions in the Hirshfeld analysis.

#### 3.1.2 Intermolecular contacts in I3OG crystals revealed by Hirshfeld analysis.

To contextualize the crystal packing of I3OG, we first computed and visualized its Hirshfeld surface. Table 1 quantifies the surface metrics and contact percentages, while Fig 2 displays the corresponding maps (dᵢ, dₑ, d_norm_, fragment patches, shape index, and curvedness).

**Table 1:** Hirshfeld surface metrics and fingerprint contact contributions (%) for I3OG.

Interaction Mode	Minimum	Mean	Maximum
d_*norm*_	−0.7461	0.4408	1.5878
d_i_	0.6392	1.6778	2.4636
d_e_	0.7011	1.6897	2.5084
Shape Index (SI)	−0.9925	0.1724	0.9996
Curvedness (Cr)	−4.0100	−1.0181	0.3573
Fingerprint% via total surface area for closed contact between atoms inside and outside the surface			
	Outside Atom%		
Inside Atoms	C	H	O
C	5.9	6.6	3.4
H	5.7	27.7	24.8
O	3.1	17.7	5.1

As summarized in Table 1, the Hirshfeld metrics reveal pronounced close contacts: the d_norm minimum −0.746 (with d_i_/d_e_ minima ≈ 0.64/0.70 Å) flags red hotspots shorter than the vdW sum, while the shape index range (~−1 to +1) and the low mean curvedness (−1.02) indicate extended, relatively flat patches suitable for π-stacking alongside H-bonding sites [31]. The fingerprint decomposition in Table 1 shows H···H = 27.7%, but the dominant directional interactions are O···H/H···O = 42.5% (24.8% + 17.7%), consistent with a strong hydrogen-bond network; hydrophobic stabilization is moderate via C···H/H···C = 12.3%, with minor C···C (5.9%) and C···O/O···C (≈6.5%) contributions.

**Fig 4:**
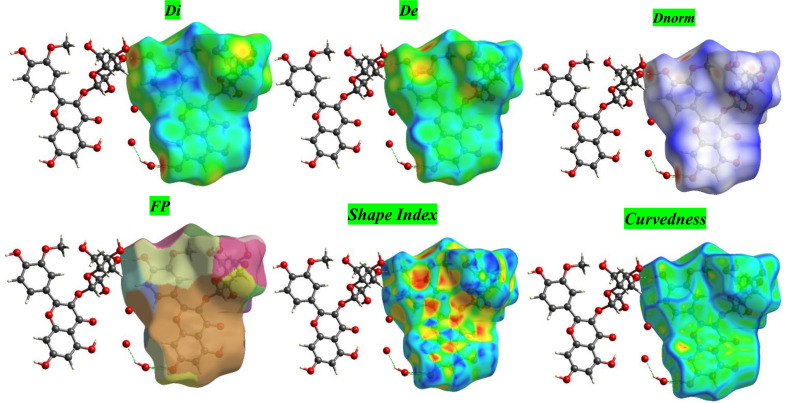
Hirshfeld surface maps of isorhamnetin-3-O-glucoside (I3OG): dᵢ, dₑ, d_norm_, fragment patches, shape index, and curvedness.

Fig 4 compiles six complementary Hirshfeld surfaces for I3OG. The dᵢ and dₑ maps locate the nearest internal and external neighbors; localized blue/cyan patches around phenolic and sugar O-H groups mark the shortest approaches. The d_norm_ surface gathers these into red hotspots contacts shorter than the vdW sum identifying the main H-bond nodes that knit adjacent molecules [32]. The FP (fragment patches) view segments the surface by contact type and visually confirms the dominance of O···H/H···O and H···H interactions. The shape index shows complementary red/blue triangular motifs on the flavonoid faces, diagnostic of π-π stacking, while the predominantly green curvedness map with limited blue ridges indicates extended flat regions separated by edges. Together, the six maps depict a surface that blends directional hydrogen bonding with aromatic stacking and moderate hydrophobic contacts, consistent with the packing features inferred from the quantitative metrics.

**Fig 5:**
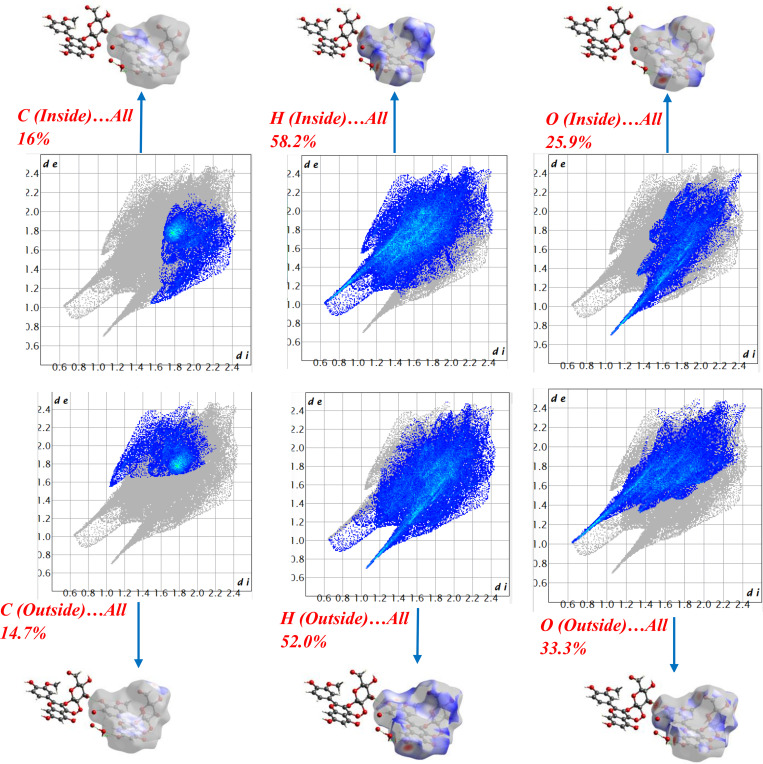
Hirshfeld surface of isorhamnetin-3-O-glucoside: 3D views (d_norm_) and 2D fingerprint plots resolved by atom type (C, H, O).

Fig 5 presents Hirshfeld fingerprint plots resolved by atom identity (inside vs. outside). Inside the surface, H atoms dominate the contacts (58.2%), followed by O (25.9%) and C (16.0%); for outside atoms the shares are H 52.0%, O 33.3%, and C 14.7%. The sharp spikes at low d_i_/d_e_ in the O(in)/H(out) and H(in)/O(out) maps diagnose directional O···H/H···O hydrogen bonds as the principal motif, in line with Table 1 and the red hotspots on d_norm_. The broader wings in the H-resolved maps indicate numerous H···H contacts, whereas the smaller carbon fractions point to a secondary hydrophobic component (C···H/H···C) rather than dominant face-to-face π-π stacking. Overall, Fig 5 shows a hydrogen-bond-led packing network complemented by moderate hydrophobic interactions.

### 3.2 Molecular docking

Molecular docking offers a mechanistic bridge between chemical structure and target modulation by predicting low-energy poses and the noncovalent interaction network within a protein active site. For Isorhamnetin-3-O-glucoside (I3OG) a polyphenolic glycoside with multiple H-bond donors/acceptors and an extended π system docking is particularly informative because it tests whether the flavonoid core and sugar hydroxyls can cooperatively engage the polar access channel and the heme-proximal pocket of inducible nitric-oxide synthase (iNOS). We selected the oxygenase domain from mouse (PDB 3E6T, 2.50 Å) and human (PDB 3E7G, 2.20 Å) to capture species-conserved recognition features and ensure translational relevance [5,26]; both entries contain the catalytic heme prosthetic group and co-bound reference inhibitors, which permits grid definition around the native ligand and preservation of the correct Fe-heme environment. Pharmacologically, iNOS inhibition is a validated anti-inflammatory strategy because pathological NO overproduction drives oxidative stress and tissue injury; thus, evaluating I3OG against these two high-quality structures tests whether a natural antioxidant scaffold can sterically and electronically complement the iNOS active site.

**Fig 6:**
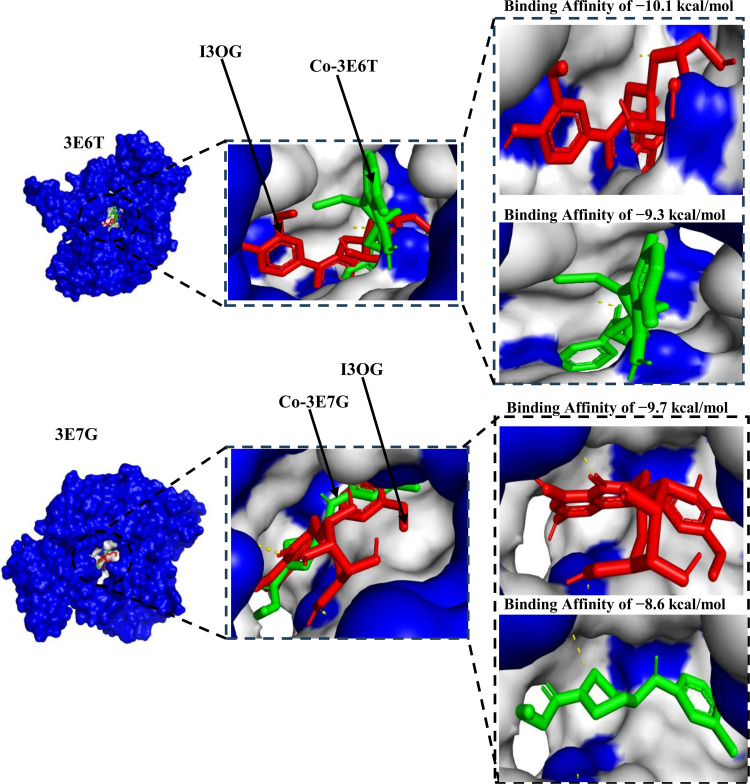
Comparative binding poses of Isorhamnetin-3-O-glucoside and co-crystallized reference inhibitors in the murine (3E6T) and human (3E7G) iNOS oxygenase domains.

As shown in Fig 6, panel A (3E6T) and panel B (3E7G) display the superposed docking poses of Isorhamnetin-3-O-glucoside (I3OG, red) and the corresponding co-crystallized reference inhibitors Co-3E6T and Co-3E7G (green) within the solvent-accessible surface of the iNOS oxygenase domains (blue, pocket in grey). In both species, I3OG closely follows the hydrophobic access channel occupied by the native ligands, with its flavonoid core buried deeper toward the heme-proximal cleft, while the glucose moiety projects toward the pocket entrance where additional polar contacts can be formed [33]. This overlay is consistent with the hydrogen-bond/hydrophobic interaction fingerprints of the co-crystallized inhibitors described in S1 and S2 Figs, and indicates that I3OG can effectively mimic the canonical pharmacophoric anchor of murine and human iNOS. The comparable predicted binding affinities (−10.1 and −9.7 kcal·mol^−1^ for I3OG vs −9.3 and −8.6 kcal·mol^−1^ for Co-3E6T and Co-3E7G, respectively) further support the ability of I3OG to stably occupy the catalytic pocket in both orthologs.

**Fig 7:**
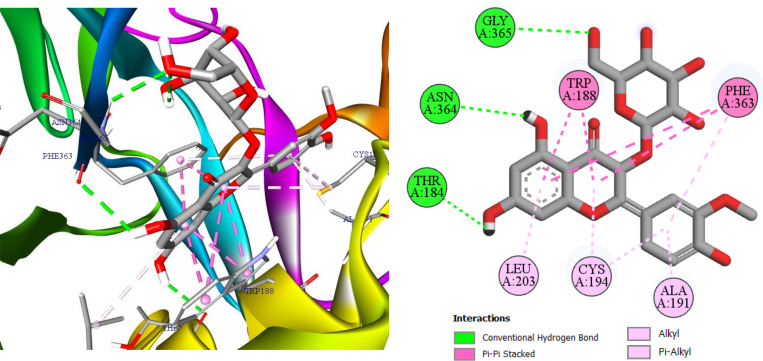
I3OG in iNOS (mouse, 3E6T): 3D/2D visualization.

**Fig 8:**
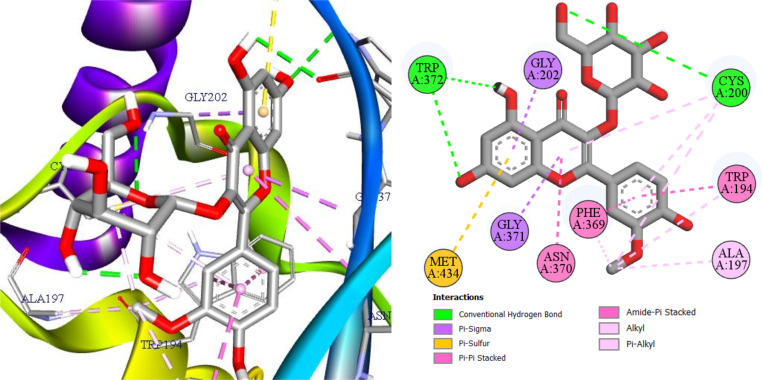
I3OG in iNOS (human, 3E7G): 3D/2D visualization.

To account for the docking scores (−10.1 kcal/mol with 3E6T and −9.7 kcal/mol with 3E7G), Figs 7 and 8 reveal a coherent interaction network that explains pose stability within the iNOS oxygenase pocket. In Fig 7 (3E6T), three conventional hydrogen bonds with Gly365, Asn364, and Thr184 anchor the ligand’s orientation in the polar channel and cooperate with four π-π stacking contacts two with Trp188 and two with Phe363 to bury the aromatic core and reinforce shape complementarity [34,35]; these are complemented by five alkyl/π-alkyl contacts, providing hydrophobic support that lowers the desolvation cost. In Fig 8 (3E7G), the ligand retains three conventional hydrogen bonds (two with Trp372 and one with Cys200), together with three π-π stacking interactions involving Phe369, Asn370, and Trp194, two π-σ contacts with Gly202 and Gly371, and a π-sulfur contact with Met434; six additional alkyl/π-alkyl contacts further tighten hydrophobic burial [36–38]. This balance of hydrogen bonding, aromatic stacking, and hydrophobic contacts across both structures explains the slight advantage for 3E6T and supports realistic shape-electrostatic complementarity for the ligand, motivating follow-up explicit-solvent MD and MM/GBSA to verify pose stability and estimate binding free energy.

### 3.3 Molecular dynamics simulation

Molecular dynamics (MD) is the critical stress-test that turns a static docking pose into a time-resolved, solvent-aware assessment of binding stability. Across a 100-ns trajectory, four standard readouts provide complementary evidence: the ligand RMSD (pose persistence relative to the protein frame), the protein backbone RMSF (local flexibility of residues surrounding the pocket), the time-series of hydrogen bonds (polar anchoring and persistence), and the protein’s radius of gyration, Rg (global compactness). Together they verify whether the docked pose for I3OG, is both kinetically stable and structurally sensible before proceeding to MM/GBSA [39–41].

**Fig 9:**
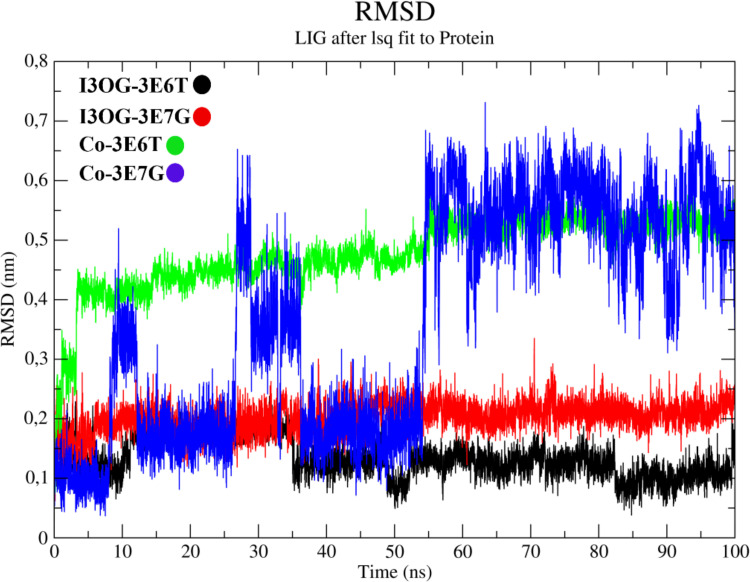
Comparative ligand RMSD profiles of Isorhamnetin-3-O-glucoside and co-crystallized inhibitors in murine (3E6T) and human (3E7G) iNOS oxygenase domains.

**Fig 10:**
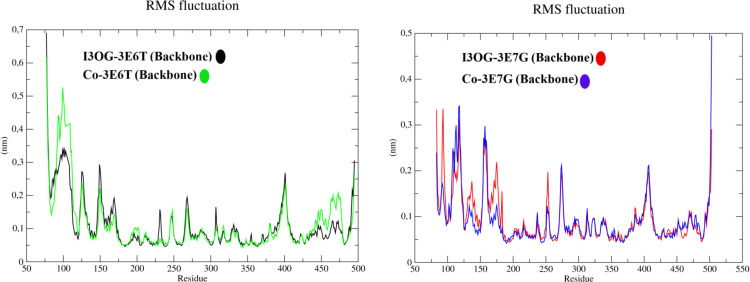
Backbone RMSF of murine and human iNOS oxygenase domains in complex with Isorhamnetin-3-O-glucoside and co-crystallized inhibitors.

Fig 9 (ligand RMSD after least-squares fit to the protein) compares the conformational stability of I3OG with that of the co-crystallized reference inhibitors in both iNOS isoforms. In all four systems, the ligands remain confined within the catalytic cleft over 100 ns with no sign of progressive drift or unbinding, but the amplitude of the fluctuations differs markedly. I3OG exhibits low RMSD values in both murine (I3OG-3E6T, black) and human (I3OG-3E7G, red) complexes, generally below ~0.25 nm, indicating that the docked poses are well preserved [42]. By contrast, the native ligands in Co-3E6T (green) and Co-3E7G (blue) populate higher RMSD basins (~0.4–0.6 nm), with the human co-crystal showing a clear shift to a more mobile regime in the second half of the trajectory. Thus, I3OG samples a tighter conformational envelope than the co-crystallized inhibitors, particularly in the murine enzyme, consistent with the overlap and interaction patterns highlighted in Figs 6–8, S1 and S2 Fig. Fig 10 (backbone RMSF) further shows that the global flexibility of both oxygenase domains remains low (~0.05–0.20 nm) and largely superimposable between I3OG-bound and co-crystal-bound simulations, confirming that neither ligand perturbs the overall fold. In 3E6T, however, I3OG slightly attenuates fluctuations at the N- and C-termini and in some loop regions flanking the binding site compared with Co-3E6T, whereas in 3E7G the RMSF traces of I3OG-3E7G and Co-3E7G are almost indistinguishable and the main peaks are confined to solvent-exposed loops, suggesting that I3OG preserves the native dynamic landscape of the human isoform [43–45].

**Fig 11:**
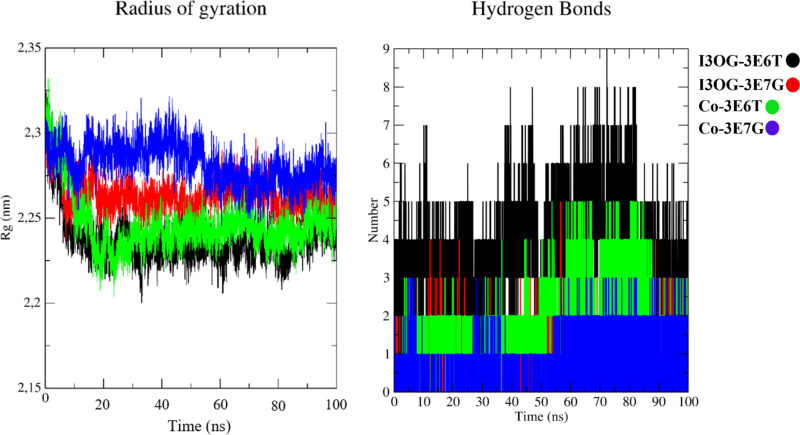
Radius of gyration and ligand–protein hydrogen bonds for Isorhamnetin-3-O-glucoside and co-crystallized inhibitors in murine and human iNOS complexes.

In Fig 11, the radius of gyration remains nearly constant for all complexes (≈2.23–2.30 nm), indicating stable global compactness without unfolding, with the I3OG-3E6T complex being slightly more compact than its counterparts. The hydrogen-bond profiles reveal a clear hierarchy in polar engagement: I3OG-3E6T sustains the densest and most persistent network (typically 4–6 simultaneous H-bonds), Co-3E6T and I3OG-3E7G display intermediate patterns (≈1–3 H-bonds), and Co-3E7G forms the sparsest contacts, often limited to one or two H-bonds.

Overall, these descriptors derived from the molecular dynamics (MD) simulations converge to show that I3OG binds with a stability comparable to that of the native inhibitors in both orthologs, and achieves particularly favorable anchoring in the murine oxygenase domain by combining restricted ligand motion, minimally perturbed backbone dynamics, preserved global compactness, and an enhanced hydrogen-bonding network.

### 3.4 MM/GBSA free-energy analysis

**Fig 12:**
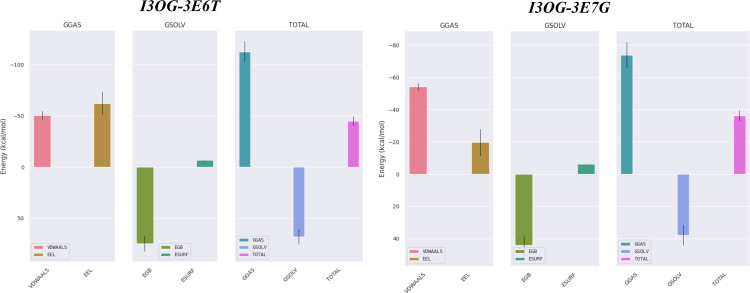
MM/GBSA binding free-energy decomposition (GGAS, GSOLV, TOTAL) for I3OG in complexes 3E6T and 3E7G (100 snapshots, 60-100 ns).


MM/GBSA complements docking and MD by estimating the binding free energy from equilibrated MD frames while decomposing the driving forces into van der Waals and electrostatics in the gas phase (ΔG_gas_) and the polar/non-polar solvation response (ΔG_solv_ = ΔE_GB_ + ΔE_surf_). Using 100 snapshots extracted from the 60–100 ns window to ensure well-equilibrated sampling, Fig 12 shows that both complexes are favorable in water (negative ΔG_bind_).

$$\Delta G_{\text{bind}\;}=\Delta G_{\text{gas}\;}+\Delta G_{\text{solv}\;}=\left(\Delta E_{\text{vdW}\;}+\Delta E_{\text{elec}\;}\right)+\left(\Delta E_{\text{GB}}+\Delta E_{\text{surf}\;}\right)\left(kcal\cdot {mol}^{-\text{1}}\right)$$

For I3OG-3E6T, the interaction is strongly driven by gas-phase terms (ΔE_vdW_ ≈ −50.5 kcal/mol and ΔE_elec_ ≈ −62.2 kcal/mol; ΔG_gas_ ≈ −112.7), partly offset by a polar desolvation penalty (ΔE_GB_ ≈ +74.6) with a small non-polar gain (ΔE_surf_ ≈ −6.8), yielding ΔG_bind_ ≈ −44.9 ± 3.9 kcal/mol (SEM ≈ 0.44) [46]. For I3OG-3E7G, the pattern is similar but weaker electrostatics (ΔE_vdW_ ≈ −54.2; ΔE_elec_ ≈ −19.7; ΔG_gas_ ≈ −73.9) and a smaller polar penalty (ΔE_GB_ ≈ +44.2; ΔE_surf_ ≈ −6.4) lead to ΔG_bind_ ≈ −36.1 ± 3.7 kcal/mol (SEM ≈ 0.31). Consistently, MM/GBSA analysis of the co-crystallized reference inhibitors yielded ΔG_bind_ ≈ −43.98 kcal·mol^−1^ for Co-3E6T and ΔG_bind_ ≈ −36.74 kcal·mol^−1^ for Co-3E7G (S3 Fig), placing I3OG within the same binding free-energy window as the native ligands in both isoforms. Thus, both proteins stabilize I3OG, but 3E6T is favored by ~9 kcal/mol because its stronger gas-phase attraction especially electrostatics more than compensates its larger desolvation cost. This energetic profile is fully consistent with the MD readouts (lower ligand RMSD and denser H-bonding in 3E6T), reinforcing the conclusion that I3OG binds more stably to 3E6T.

**Fig 13:**
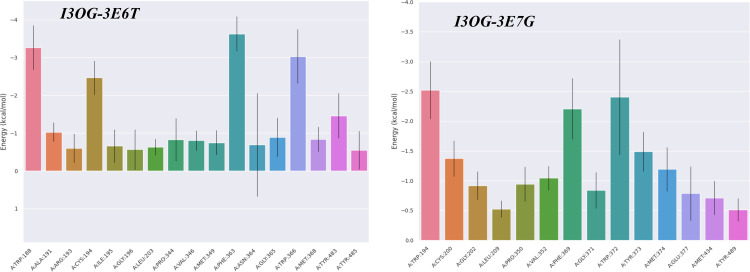
MM/GBSA per-residue binding free-energy contributions (ΔG_bind_; mean ± SEM) for I3OG in complexes 3E6T and 3E7G (100 snapshots, 60-100 ns).

Fig 13 presents the residue-wise MM/GBSA decomposition from 100 snapshots taken between 60−100 ns, mapping the “hot spots” that anchor I3OG in the active site; residues with per-residue contributions < −1 kcal/mol are considered stabilizing. In I3OG-3E6T, Trp188, Cys194, Trp366, and Phe363 show < −2 kcal/mol and thus act as the primary anchors fully consistent with the docking contacts and additional pocket residues just below −1 kcal/mol form a hydrophobic belt that helps keep the ligand seated. In I3OG-3E7G, three key residues dominate (Trp194, Phe369, Trp372), each < −2 kcal/mol; notably, Trp372 was flagged in docking for two conventional hydrogen bonds, which agrees with the MD hydrogen-bond trace (Fig 11) where two H-bonds persist from ~60 ns to the end of the run. For the co-crystallized reference ligands, the per-residue MM/GBSA profiles (S4 and S5 Figs) display a highly similar hot-spot distribution, with Trp188, Cys194, Pro344, Val346, Arg193 and Phe363 acting as major anchors in Co-3E6T and Trp194, Cys200, Pro350, Val352 and Phe369 dominating in Co-3E7G, indicating that I3OG engages essentially the same anchoring network as the native inhibitors. Overall, Fig 13 confirms that the ligand is well stabilized in the active site of both proteins, with deeper hot spots in 3E6T, in line with its more favorable ΔG_bind_ and MD stability readouts.

**Fig 14:**
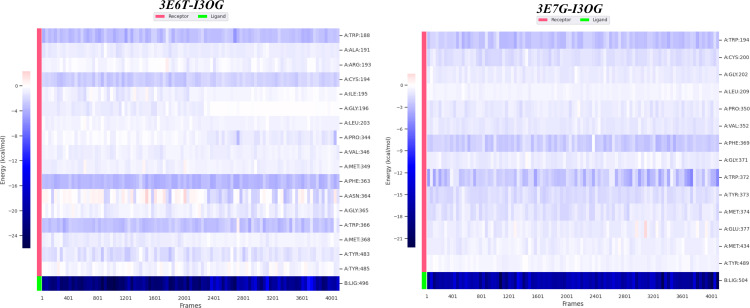
Time-resolved heatmaps of per-residue MM/GBSA interaction energies over 60-100 ns for I3OG bound to 3E6T (left) and 3E7G (right).

Fig 14 is a time-resolved heat map of the per-residue MM/GBSA interaction energies over the 60–100 ns window (blue = favorable/negative; pale = weak or transient). In I3OG-3E6T, the darkest, most continuous bands sit on Phe363 (dominant hotspot) and Trp366, with persistent contributions from Trp188 and Cys194 exactly the anchors highlighted by Fig 13 while loop residues show intermittent, lighter stripes indicative of fleeting contacts. In I3OG-3E7G, Trp372 is the chief hotspot, followed by Phe369 and Trp194; the sustained dark-blue signal on Trp372 agrees with docking (two conventional H-bonds) and with the MD H-bond trace in Fig 11, where two hydrogen bonds persist from ~60 ns to the end. The bottom LIG (I3OG) row remains strongly negative throughout in both panels, confirming a stable net attraction. Overall, Fig 14 corroborates Fig 13: the key hotspots stay engaged across the trajectory, with more continuous strong contacts in 3E6T, consistent with its more favorable ΔG_bind_.

### 3.5 Principal-component energy landscape analysis

Principal component analysis (PCA) compresses the slow, collective motions sampled by MD into a few coordinates; projecting the trajectory onto PC1-PC2 and transforming occupancy into a free-energy surface G = - kB TlnP identifies the metastable states and the barriers between them.

**Fig 15:**
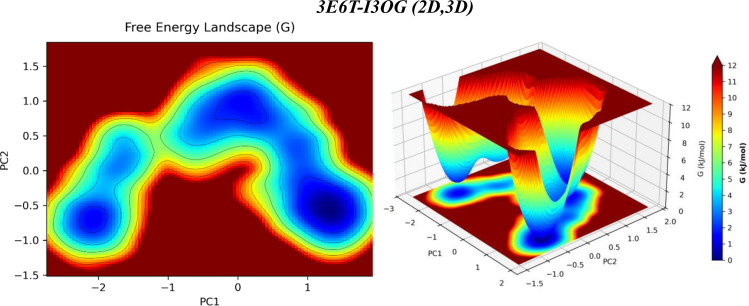
PCA-derived free-energy landscape (PC1 vs PC2) for the 3E6T-I3OG complex from the 60-100 ns MD window (2D heat map, left; 3D surface, right; color bar: G, kJ·mol^−1^).

In Fig 15, the 3E6T-I3OG landscape shows two to three neighboring wells along PC1, separated by low barriers of only a few kJ·mol^−1^ (per the 0–12 kJ·mol^−1^ color bar), indicating small-amplitude pocket “breathing” and micro-rearrangements around a single bound state; the minima are relatively narrow and transitions remain confined near the active site, a signature of local stability.

**Fig 16:**
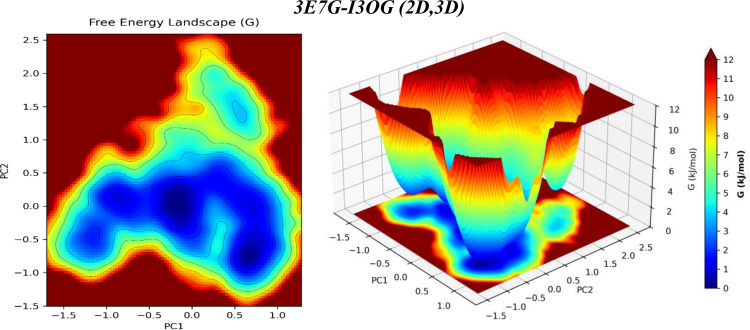
PCA-derived free-energy landscape (PC1 vs PC2) for the 3E7G-I3OG complex from the 60-100 ns MD window (2D heat map, left; 3D surface, right; color bar: G, kJ·mol^ −1^).

By contrast, Fig 16 for 3E7G-I3OG presents one broad, comparatively flat valley that spreads mainly along PC2; this diffuse basin reflects a more compliant pocket and a wider entropic distribution of conformations, with less energetic focusing than in 3E6T. Consistently, the free-energy landscapes of the co-crystallized reference ligands (S6 and S7 Figs) display the same qualitative behavior, with Co-3E6T sampling a compact, funnel-like single basin and Co-3E7G exploring a broader, multi-minima low-energy region, indicating that the murine oxygenase domain is intrinsically more dynamically focused than the human ortholog and that the I3OG simulations faithfully reproduce the dynamical signatures of the native inhibitors. Read together, the PCA/FEL therefore supports the overall MD picture: 3E6T is dynamically more stabilized, with a more concentrated set of deep minima, whereas 3E7G remains more plastic and explores a broader low-energy region. This resolves the earlier inconsistency and aligns with our other metrics tighter ligand RMSD, lower pocket Cα-RMSF, more persistent H-bonding, and a more favorable MM/GBSA ΔG_bind_ for 3E6T.

### 3.6 Density functional theory analysis

To complement docking, MD and MM/GBSA and to rationalize the noncovalent binding preferences at the electronic-structure level, we performed gas-phase DFT calculations on the isolated ligands [33]. Evaluating frontier molecular orbitals and global reactivity descriptors in the gas phase is a standard practice because these quantities are intrinsic molecular properties that primarily reflect the internal π-conjugation pattern and the balance between electron-donating and electron-accepting groups. Solvent or protein environments mainly induce nearly rigid shifts of orbital energies without altering qualitative trends, whereas explicit environmental effects are already captured in our MD and MM/GBSA analyses. Gas-phase DFT is therefore appropriate here to compare the relative electronic softness, polarity and electrophilicity of I3OG versus the co-crystallized reference inhibitors and to connect these intrinsic features with their binding profiles.

**Fig 17:**
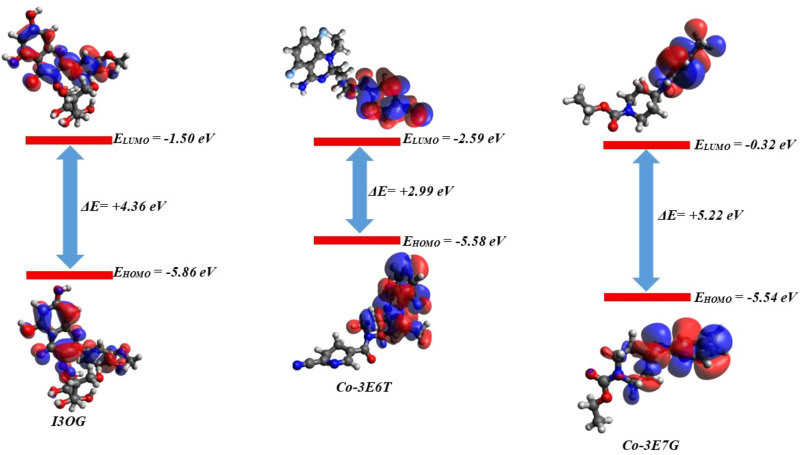
Frontier molecular orbitals and HOMO–LUMO energy level diagrams of I3OG, Co-3E6T and Co-3E7G obtained from gas-phase DFT calculations.

Table 2 summarizes the global descriptors derived from the HOMO/LUMO energies, while Fig 17 visualizes the corresponding frontier orbitals. I3OG exhibits E_HOMO_ = −5.86 eV and E_LUMO_ = −1.50 eV, giving an intermediate gap ΔEgap = 4.36 eV and a moderate hardness/softness pair (η = 2.18 eV, S = 0.229 eV ⁻ ¹). This places I3OG between the two reference ligands: Co-3E6T shows the smallest gap (2.99 eV; η = 1.50 eV; S = 0.334 eV ⁻ ¹), indicating the softest and most easily polarizable π-system, whereas Co-3E7G displays the largest gap (5.22 eV; η = 2.61 eV; S = 0.191 eV ⁻ ¹), characteristic of a harder, more electronically inert scaffold [47,48]. The chemical potential and electrophilicity index follow the same hierarchy: Co-3E6T is the most electrophilic (μ ≈ −4.09 eV, ω = 5.58 eV), I3OG occupies an intermediate regime (μ ≈ −3.68 eV, ω = 3.11 eV), and Co-3E7G is the least electrophilic (μ ≈ −2.93 eV, ω = 1.64 eV). Thus, I3OG is predicted to engage in polarization and charge-transfer interactions more readily than the human co-crystal ligand and in a manner that approaches the murine co-crystal ligand, which is fully consistent with the MM/GBSA results showing that I3OG and Co-3E6T share very similar ΔGbind values in 3E6T and both outperform their counterparts in 3E7G.

**Table 2:** DFT-derived global electronic descriptors (HOMO–LUMO energies, energy gap, hardness, softness, chemical potential and electrophilicity index) for I3OG and the co-crystallized reference ligands Co-3E6T and Co-3E7G in the gas phase.

Descriptor	I3OG (eV)	Co-3E6T (eV)	Co-3E7G (eV)
HOMO energy	−5.86	−5.58	−5.54
LUMO energy	−1.50	−2.59	−0.32
Energy gap (HOMO-LUMO)	4.36	2.99	5.22
Hardness (η)	2.18	1.495	2.61
Softness (S, eV ⁻ ¹)	0.229	0.334	0.191
Chemical potential (μ)	−3.68	−4.085	−2.93
Electrophilicity index (ω)	3.11	5.58	1.64

The spatial distribution of the frontier orbitals in Fig 17 provides a more mechanistic picture. For I3OG, both HOMO and LUMO are broadly delocalized over the conjugated flavonol core and extend onto the glucoside moiety, indicating that electron density rearrangement upon interaction can involve simultaneously the aromatic system and the polar sugar hydroxyls. This dual electronic participation matches the binding mode deduced from docking and MD, in which the aglycone π-system engages hydrophobic and π-stacking contacts deep in the pocket while the sugar hydroxyls mediate an extended hydrogen-bond network at the pocket entrance [49]. In Co-3E6T, the HOMO/LUMO are concentrated on the planar aromatic/heteroaromatic scaffold that directly faces the iNOS hydrophobic channel, in line with its strong van der Waals and electrostatic components and its pronounced electrophilicity. By contrast, Co-3E7G exhibits a larger HOMO–LUMO separation with more localized frontier orbitals, confined mainly to one end of the molecule, consistent with its higher hardness and lower electrophilicity and with the somewhat weaker and more diffuse binding pattern previously observed for the 3E7G complexes.

Overall, the gas-phase DFT analysis shows that I3OG possesses an intermediate softness and electrophilicity profile and frontier orbitals delocalized over both the aromatic core and the sugar appendage, enabling it to mimic the electronic behaviour of the murine co-crystal ligand while retaining sufficient stability. These intrinsic electronic features dovetail with the conformational stability (RMSD/RMSF), dense hydrogen-bonding network, localized free-energy minima and favorable MM/GBSA ΔG_bind_ obtained for the 3E6T complexes, thereby providing a coherent, quantum-chemical justification for the preferential stabilization of I3OG in the murine iNOS oxygenase domain.

## 4. Conclusion

This work identifies isorhamnetin-3-O-glucoside (I3OG) as a credible, mechanism-aligned scaffold for attenuating iNOS activity. Consistent docking, restrained ligand dynamics, and favorable MM/GBSA estimates converge to support stable engagement of the iNOS oxygenase domain, with stronger stabilization in the murine (3E6T) than the human (3E7G) construct and clear residue-level hot spots that rationalize this species dependence. These results argue that I3OG and closely related chromone/flavonol chemotypes merit progression beyond in silico screening. Immediate priorities are orthogonal biophysical validation (SPR/ITC), enzyme inhibition and NO-suppression assays in relevant macrophage models, and selectivity profiling against eNOS/nNOS. Given the permeability liabilities of glycosides, medicinal chemistry should explore aglycone analogs, sugar bioisosteres, and prodrug strategies while preserving the π-stacking and hydrogen-bonding motifs highlighted here. Finally, longer simulations and models incorporating full cofactor/dimer contexts will refine translatability to the human enzyme. Collectively, our data provide a quantitative blueprint for structure-guided iNOS inhibitor design and a tractable starting point for anti-inflammatory lead optimization.

## Supporting information

S1 File Figure S1. (DOCX)

S2 File Figure S2. (DOCX)

S3 File Figure S3. (DOCX)

S4 File Figure S4. (DOCX)

S5 File Figure S5. (DOCX)

S6 File Figure S6. (DOCX)

S7 File Figure S7. (DOCX)

## Abbreviations

RMSD: Root Mean Square Deviation
RMSF: Root Mean Square Fluctuation
iNOS: Inducible nitric oxide synthase
ADME: Absorption, Distribution, Metabolism, and Excretion
I3OG: Isorhamnetin-3-O-Glucoside.
