# Supplementary material for: Elucidating the isorhamnetin-3-O-glucoside-iNOS interaction via molecular dynamics and Hirshfeld surface analyses
Source: PLoS One. 2025 Dec 19;20(12):e0339357. doi: 10.1371/journal.pone.0339357 (PMC12716702; doi:10.1371/journal.pone.0339357)
Supplement: S1 File — (DOCX) [file pone.0339357.s001.docx]

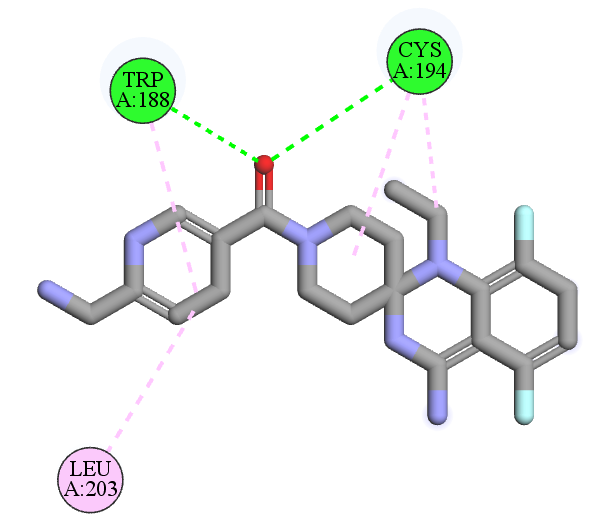

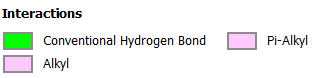

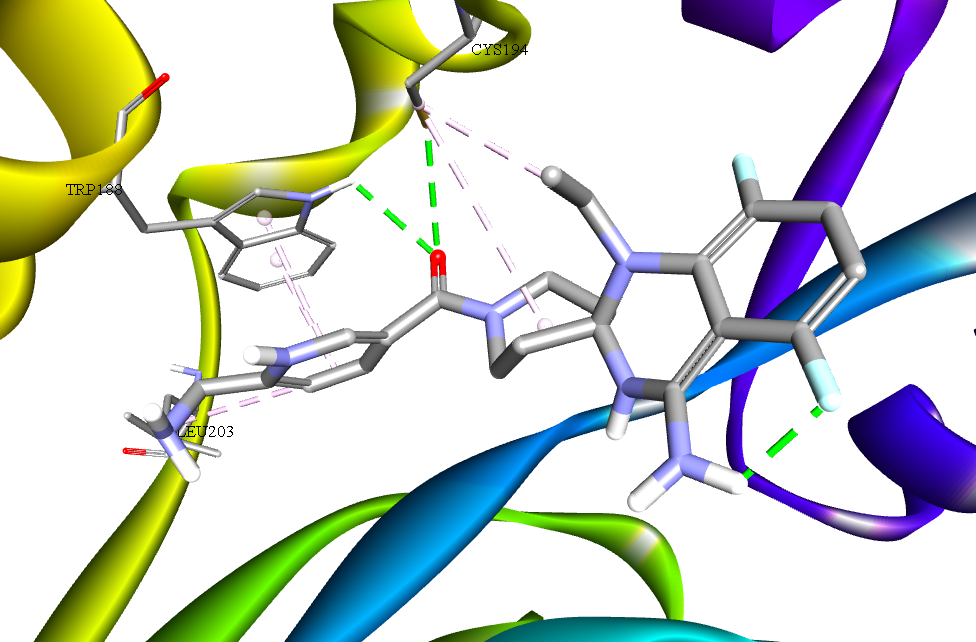


***Co-3E6T***

**Figure S1.** Reference binding mode of the co-crystallized inhibitor in the murine iNOS active site (PDB ID 3E6T). Left: 3D view of the co-crystallized inhibitor (Co-3E6T) bound to the oxygenase domain of murine iNOS. Right: 2D interaction diagram. The central carbonyl group of the inhibitor acts as a hydrogen-bond acceptor for TRP188 and CYS194 (green dashed lines), while the aromatic and alkyl moieties establish π-alkyl and alkyl contacts with CYS194 and LEU203 (pink dashed lines). This dual hydrogen-bond/hydrophobic clamp defines the pharmacophoric anchor of the native ligand at the mouth of the catalytic pocket and was used as a reference for docking and MD analyses of I3OG.
