# Supplementary material for: Elucidating the isorhamnetin-3-O-glucoside-iNOS interaction via molecular dynamics and Hirshfeld surface analyses
Source: PLoS One. 2025 Dec 19;20(12):e0339357. doi: 10.1371/journal.pone.0339357 (PMC12716702; doi:10.1371/journal.pone.0339357)
Supplement: S2 File — (DOCX) [file pone.0339357.s002.docx]

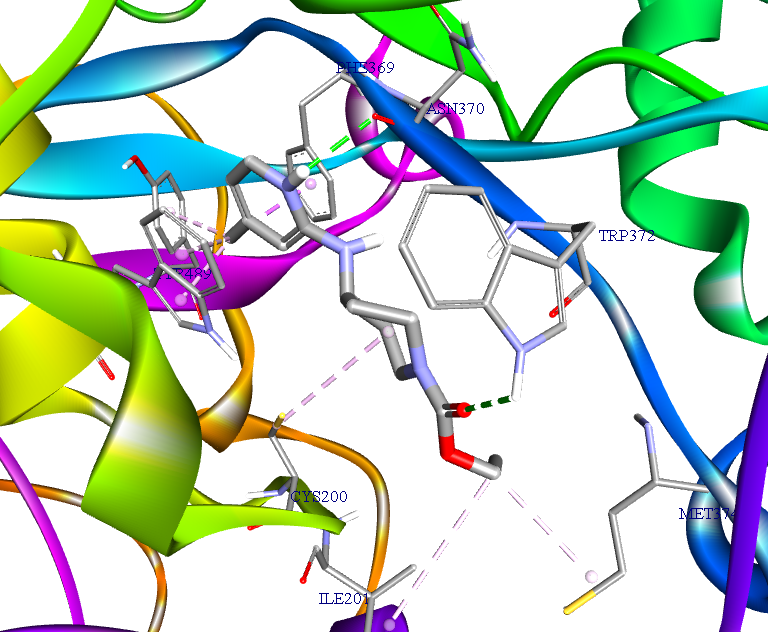

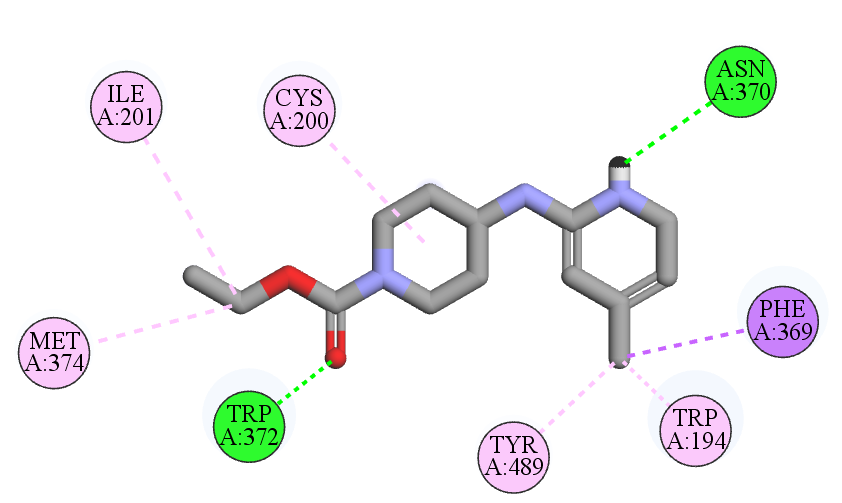

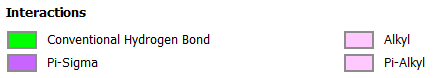


***Co-3E7G***

**Figure S2.** Reference binding mode of the co-crystallized inhibitor in the human iNOS active site (PDB ID 3E7G). Left: 3D view of the co-crystallized inhibitor (Co-3E7G) bound to the oxygenase domain of human iNOS. Right: 2D interaction diagram. The inhibitor adopts an extended conformation along the access channel, where its carbonyl group and adjacent heteroatoms form conventional hydrogen bonds with TRP372 and ASN370 (green dashed lines). The aromatic rings and aliphatic linker establish a dense hydrophobic network of alkyl, π-alkyl and π-sigma contacts with CYS200, ILE201, MET374, PHE369, TRP194 and TYR489 (pink and purple dashed lines). This H-bond/hydrophobic interaction pattern defines the native pharmacophore of the human enzyme and served as a reference for docking and MD comparisons with I3OG.
