# Supplementary material for: Elucidating the isorhamnetin-3-O-glucoside-iNOS interaction via molecular dynamics and Hirshfeld surface analyses
Source: PLoS One. 2025 Dec 19;20(12):e0339357. doi: 10.1371/journal.pone.0339357 (PMC12716702; doi:10.1371/journal.pone.0339357)
Supplement: S5 File — (DOCX) [file pone.0339357.s005.docx]

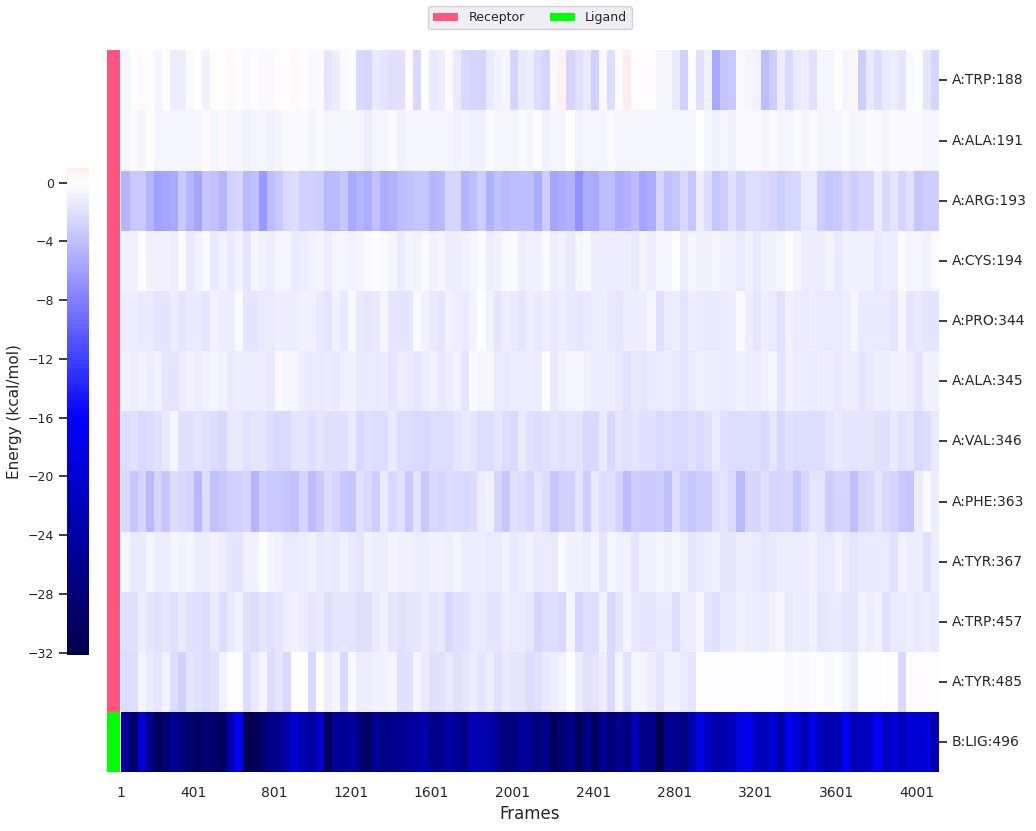

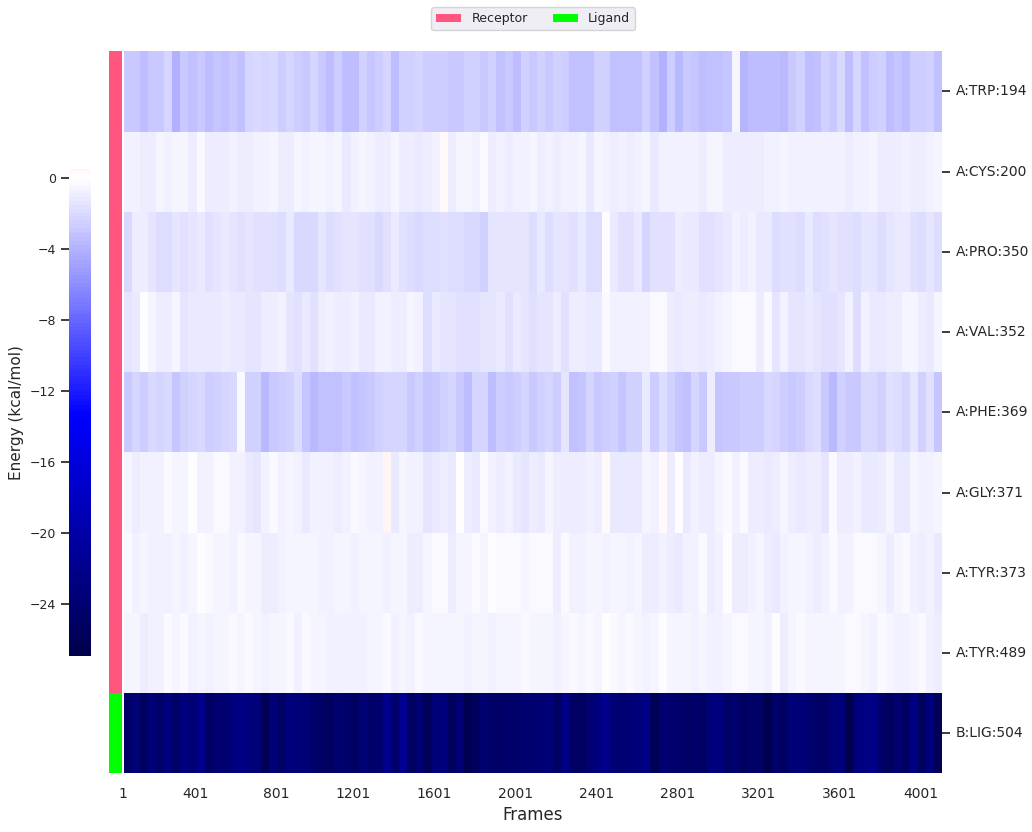


***Co-3E6T***

***Co-3E7G***

**Figure S5.** Time-resolved MM/GBSA heat maps of residue-wise receptor–ligand interaction energies for the co-crystallized inhibitors Co-3E6T and Co-3E7G.
