# Supplementary material for: Elucidating the isorhamnetin-3-O-glucoside-iNOS interaction via molecular dynamics and Hirshfeld surface analyses
Source: PLoS One. 2025 Dec 19;20(12):e0339357. doi: 10.1371/journal.pone.0339357 (PMC12716702; doi:10.1371/journal.pone.0339357)
Supplement: S6 File — (DOCX) [file pone.0339357.s006.docx]

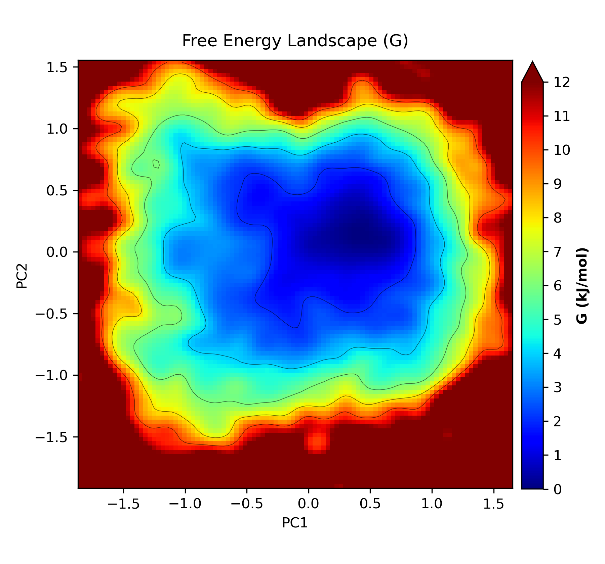

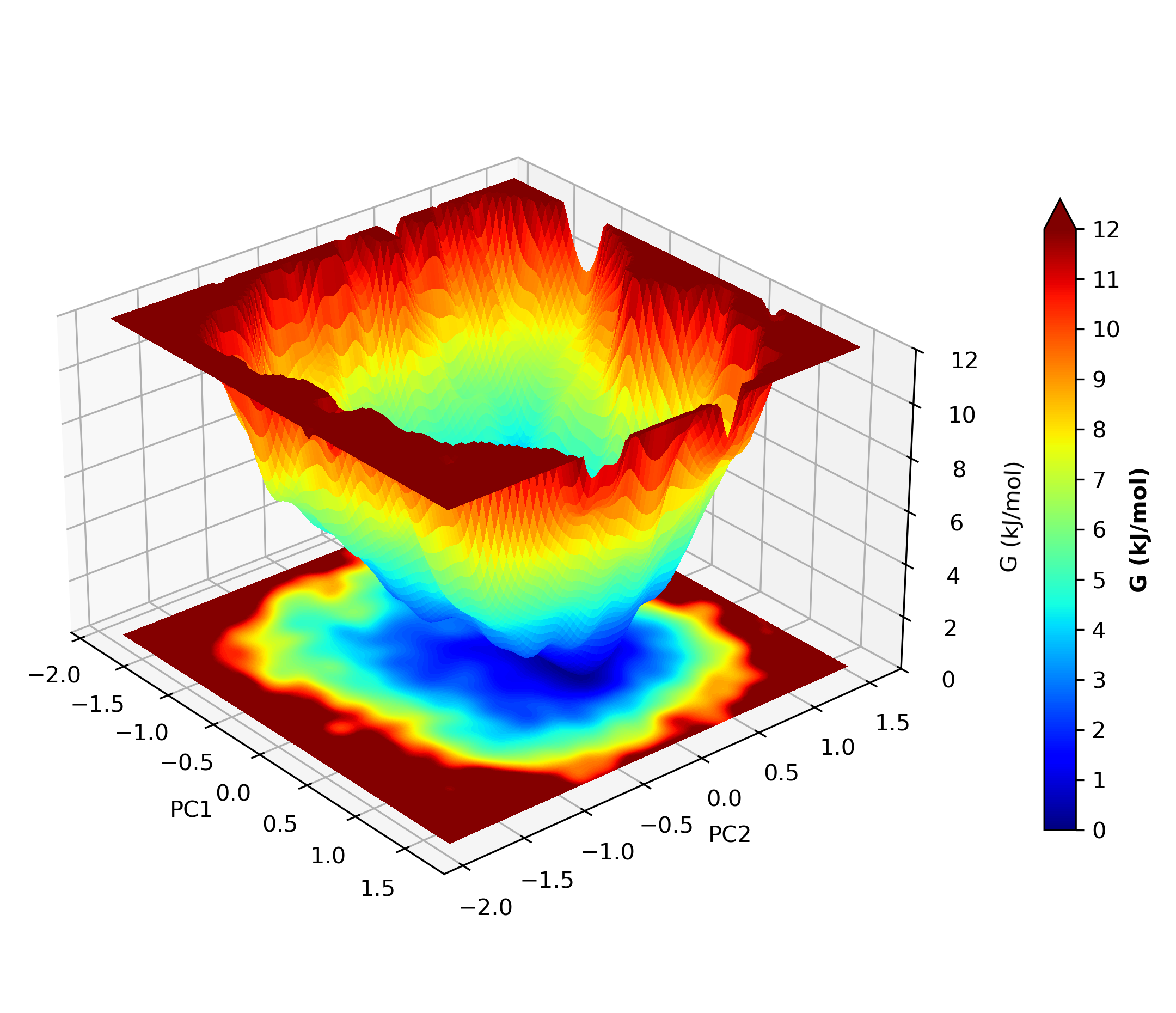


***3D***

***2D***

***Co-3E6T***

**Figure S6.** Two- and three-dimensional PCA free-energy landscapes (PC1 vs PC2) for the Co-3E6T complex.
